# The thymus in myasthenic patients: correlation between mediastinal CT imaging and histopathological findings

**Published:** 2012

**Authors:** GA Popa, C Scheau, EM Preda, IG Lupescu

**Affiliations:** Department of Radiology and Medical Imaging, Fundeni Clinical Institute, Bucharest, Romania

**Keywords:** Thymus, myasthenia gravis, computed tomography (CT), computed tomography = CT

## Abstract

Purpose: To correlate computed tomographic (CT) appearance of the thymus with results from histologic examination of thymic tissue in myasthenic patients who underwent thymectomy.

Materials and methods: A retrospective study, based on case series report, between January 2000 and December 2010 on 247 patients with generalized myasthenia gravis or myasthenic syndrome explored by CT using a specific protocol, in the Radiology and Medical Imaging Department of Fundeni Clinical Institute. All subjects in the study were operated and had a histologic evaluation.

Results: CT examinations of these 247 subjects showed remaining thymic mass, remnant of thymic tissue, fatty infiltration of the thymus and tumoral thymus or focal thymic mass. The results of histologic examination showed normal thymus, thymic lymphoid follicular hyperplasia, thymic atrophy, fatty infiltration and thymoma.

Conclusion: It was a 100% correlation between CT examinations and intra-operative findings regarding the presents of focal thymic masses in our study. In the diagnostic of focal thymic mass, the only inter-disciplinary correlation is between radiological and macroscopic aspects. CT examination has a limited value in differential diagnosis between thymic lymphoid follicular hyperplasia and thymoma.

## Background:

The thymus is a heterogeneous admixture of lymphoid and epithelial elements located in the superior and anterior portions of the mediastinum. It is an integral part of the immune system.

Its shape is partially determined by adjacent structures. Posteriorly, it molds itself against the trachea, the left brachiocephalic vein, the aortic arch and its branches, and the pericardium covering the ascending aorta and the pulmonary trunk. Anteriorly, the sternum and the antero-medial margins of the lungs cover it. It is a bilobed structure, with the left lobe usually extending further inferiorly than the right. The two lobes usually make contact superiorly near the midline and then diverge inferiorly. Each lobe is enclosed in its own thin fibrous capsule; delicate septa divide the thymic lobes into irregular lobules in the younger subjects [**1**].

## Purpose:

The purpose of the paper is to correlate computed tomographic aspect of the thymus with the results from histologic examination of thymic tissue in myasthenic patients who underwent thymectomy and to describe different CT aspects in patients with thymic lymphoid follicular hyperplasia and thymoma.

This report details our experience in the evaluation of thymic diseases using CT since 1984. We took into consideration 247 patients examined by plain chest radiography and computed tomography, in our department, spanning a period of over 10 years. This method is considered helpful in the detection of thymoma but had a limited value in the detection of thymic hyperplasia because most glands with histologic evidence of lymphoid follicular hyperplasia are reported to be normal in size.

## Materials and methods:

We made a retrospective study, based on case series report, between January 2000 and December 2010. We included 247 subjects in this study, who were operated with histologic evaluation available. All the patients had passed a sequential or spiral CT scan obtained in deep-inspiration breath-hold. Sequential CT scans were performed while using a 0,5 cm collimation, contiguous throughout the thorax; we performed contiguous 0,2-0,3 cm collimation slices for densitometric characterization of micronodules. Intravenous administration of nonionic iodinated contrast media was used occasionally to delineate the thymus from the aorta, superior vena cava and pulmonary artery. We used iodinated contrast media with 350-370-400 mg iodine/ml, 1 ml/kg, with a flow rate of 3 ml/sec. We analyzed the global variation in shape, size and density of the normal thymus and visualized different CT aspects in patients with following histological entities: normal thymus, thymic lymphoid follicular hyperplasia, thymic atrophy, fatty infiltration and thymoma.

## Results:

All the patients from our study had associated myasthenia gravis or myasthenic syndrome.

In this study, female patients were affected two times more than male patients.

In 105 patients with remaining thymic mass evidenced by CT scan, histopathological evaluation revealed thymic hyperplasia in 88 patients (83.80%), normal thymus in 2 patients (1,90%), thymic atrophy in 7 patients (6.67%), fatty infiltration in one patient (0.96%) and thymoma in 7 patients (6.67%). In 60 patients with remnant of thymic tissue evidenced by CT scan, histopathological evaluation showed thymic hyperplasia in 23 patients (38.33%), normal thymus in 4 patients (6.67%), thymic atrophy in 31 patients (51.66%), fatty infiltration in one patient (1.67%) and thymoma in one patient (1.67%).

Tumoral thymus was revealed in 41 patients by CT scan and histological examination revealed the following entities: thymic hyperplasia in 13 patients (31.70%), thymic atrophy in one patient (2.45%) and thymoma in 27 patients (65.85%).

**Fig. 1:**
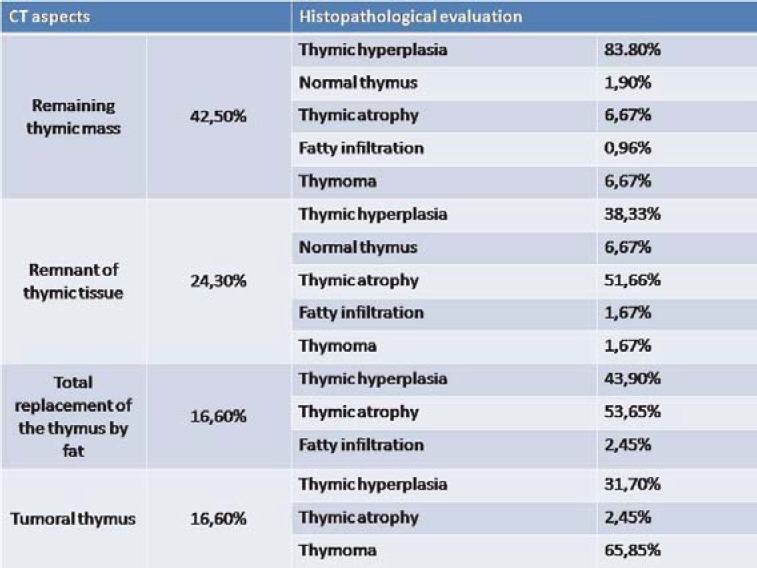
The relationship between CT results and histopathologic findings

The relationship between CT results and histopathologic findings are summarized in the table (**Fig. 1**).

**Fig. 2:**
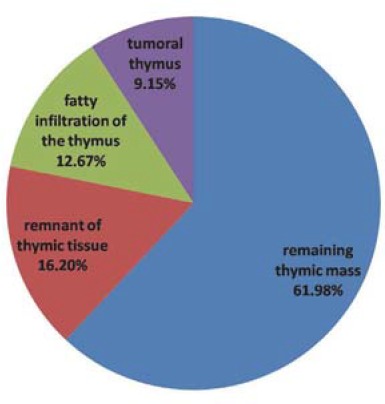
CT aspects in all patients with thymic lymphoid follicular hyperplasia

**Fig. 3:**
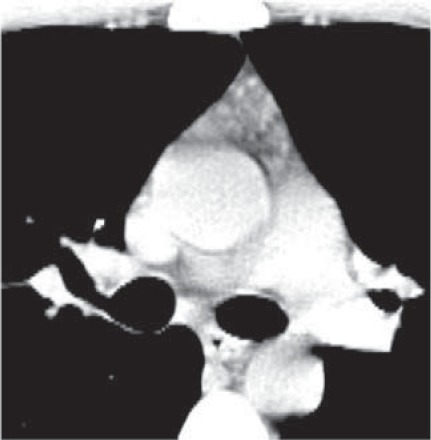
29-y/o female with myasthenia gravis; CT scan shows remaining thymic mass with early fatty infiltration, pathologic diagnosis - thymic hyperplasia

Different aspects were noted on CT scans in all 142 patients with thymic lymphoid follicular hyperplasia: remaining thymic mass (61.98%), remnant of thymic tissue (16.20%), fatty infiltration of the thymus (12.67%) and tumoral thymus (9.15%) (**Fig. 2,3**).

**Fig. 4:**
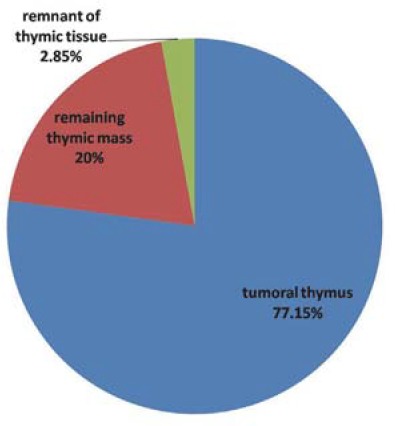
CT aspects in all patients with thymoma

**Fig. 5:**
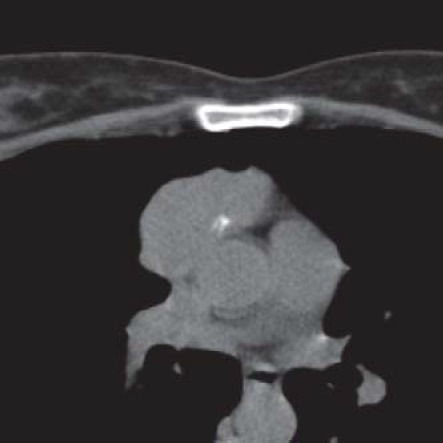
35-y/o female with myasthenia gravis; CT scan shows a tumoral mass in the prevascular space with minimal calcification; pathologic diagnosis - thymoma

In 35 patients with surgically proved thymomas, CT scan showed: tumoral thymus (77.15%), remaining thymic mass (20%) and remnant of thymic tissue (2.85%) (**Fig. 4,5**).

**Fig. 6:**
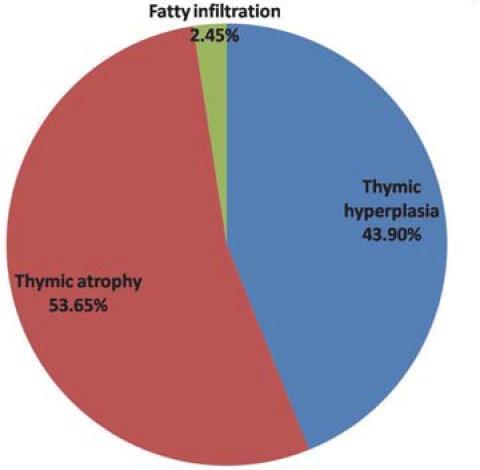
Different pathologic aspects in all patients with partial or total replacement of the thymus by fat on CT scan

In 41 patients with total replacement of the thymus by fat on CT scan, histopathological evaluation revealed: thymic atrophy in 21 patients (53.65%), thymic hyperplasia in 18 patients (43.90%) and fatty infiltration in 1 patient (2.45%) (**Fig. 6**).

## Discussions.

This report details our experience in the evaluation of thymic diseases using CT since 1984. We took into consideration 247 patients examined by plain chest radiography and computed tomography, in our department, spanning a period of over 10 years.

In our study, thymic hyperplasia most often correlates with remaining thymic mass on CT scan (83.80%), thymic atrophy with remnant of thymic tissue (51.66%) and total replacement of the thymus by fat (53.65%), and thymomas with tumoral mass in the anterior mediastinum (65.85%). Data revealed by our study are similar to those found in literature.

The thymus achieves its maximal weight between 12 and 19 years; between 20 and 60 years, regression in size occurs, together with replacement by adipose [**2**].

The CT examination is considered helpful in the detection of thymoma but had a limited value in the differential diagnosis of lymphoid follicular hyperplasia and thymoma. Computed tomography is the study of choice for evaluating disease in the anterior mediastinum [**3**].The normal thymus on CT scans varied with the patients’ age.

**Fig. 7:**
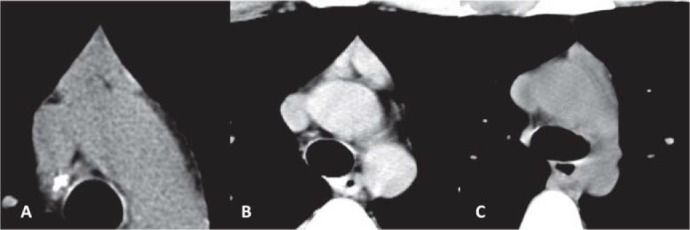
The shape of normal thymus in adults. (A) "Arrowhead" configuration, when the two lobes are confluent. 17-y/o female with myasthenia gravis; CT scan shows remaining thymic mass with density similar to muscle; pathologic diagnosis - thymic hyperplasia. (B) "Bilobed thymus" with two separate lobes. 22-y/o male with myasthenia gravis; CT scan shows diffusely enlarged thymus with higher density than the chest wall musculature; pathologic diagnosis - benign thymoma. (C) One lobe visualized. 31-y/o female with myasthenia gravis; CT scan shows remaining thymic mass; pathologic diagnosis - thymic hyperplasia [**4**].

*The shape of normal thymus in adults. *Baron individualized three configurations of the normal thymus: "arrowhead" configuration, when the two lobes are confluent; two separate lobes, the shapes may be characterized as ovoid, elliptical, triangular, or semilunar; one lobe visualized, in a small number of cases [**1**]. We visualized different CT aspects in patients with normal histological thymus appearance (**Fig. 7**) [**4**].

**Fig. 8:**
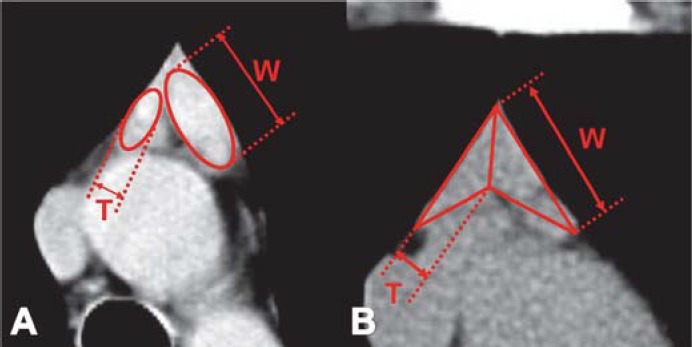
The measurement of thymic size in a bilobed and arrowhead-shaped organ. The width (W) - longest axis of the lobe as visualized on a transverse scan. The thickness (T) – the largest dimension perpendicular to the long axis of the lobe [**4**].

*The size of normal thymus in adults. *The width represents the longest axis of the lobe as visualized on a transverse scan and the thickness represents the largest dimension perpendicular to the long axis of the lobe. The width and the thickness of each lobe of the thymus were measured [**1**]. The thymus with "arrowhead" configuration, must be divided in the midline into a right and left lobe to obtain measurements of the width and the thickness of each lobe (**Fig. 8**) [**1,4**].

The width decreases in older patients, but a large variation exists within each age group. The thymus becomes narrower with increasing age, a wide variation of the thickness exist in different age groups [**1**]. The maximum thickness was of 1.8 cm in patients under 20 years old and of 1.3 cm in patients over 20 years old [**5**].

**Fig. 9:**
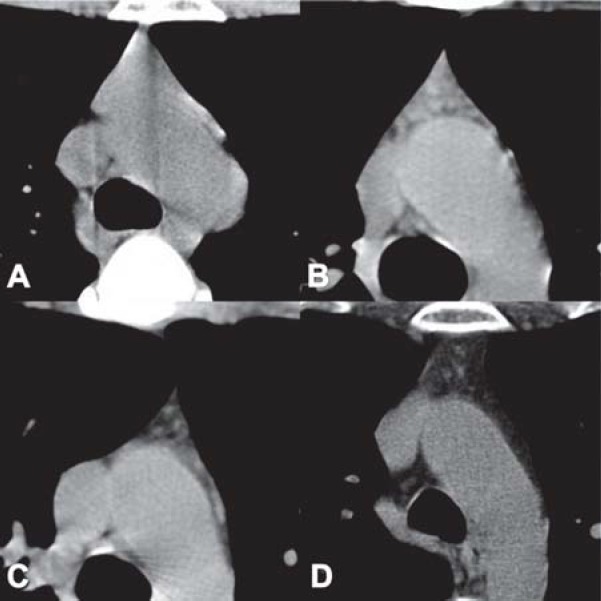
Progressive fatty infiltration of the thymus. (A) 20-y/o female with myasthenia gravis; CT scan shows remaining thymic mass with density similar to muscle; pathologic diagnosis - thymic hyperplasia. (B) 28-y/o female, with myasthenia gravis; CT scan shows remaining thymic mass with early fatty infiltration; pathologic diagnosis – thymic hyperplasia. (C) 32-y/o female with myasthenia gravis; CT scan shows remnant of thymic tissue; pathologic diagnosis - thymic atrophy. (D) 46-y/o female with myasthenia gravis; CT scan shows replacement of the thymus by fat; pathologic diagnosis - thymic atrophy [**4**].

*The density of normal thymus in adults. *The mean attenuation value of the thymus was obtained and compared with that of the chest wall muscles. The CT attenuation values decrease with aging. In patients under 25 years of age, the thymus is isodense or with a higher density than the chest wall musculature; between 25 and 49 years the thymus appears with a lower density than that of associated soft tissues because of progressive fatty infiltration; patients over 50 years of age had a thymic density approaching to the fat (**Fig. 9**) [**1,4**].

The CT scan revealed a lobular architecture of the thymus with fatty replacement visible between the lobules [**1**].

In our study, the remnant of the thymic tissue was characterized as normal if micronodules with soft-tissue attenuation were present and did not produce a focal lateral convexity along the mediastinal boundary. In literature [**6**], rounded anterior mediastinal areas of soft-tissue attenuation were considered to represent normal residual thymus rather than a small mass when they did not exceed 7 mm in diameter [**4**].

*The contours of normal thymus in adults. *To differentiate small tumors from the remnant of the thymic tissue is necessary to analyze glandular and anterior mediastinal contours. The normal thymus had rectilinear or concave contours. A well-defined convex contour is associated with a small tumor, rather than with a remnant of the thymic tissue.

*Thymic hyperplasia*. There are two distinct histologic types of thymic hyperplasia: true thymic hyperplasia and lymphoid hyperplasia, both manifest as diffuse symmetric enlargement of the thymus, so that it is difficult to distinguish between the two types on the basis of imaging findings alone [**5**].

Lymphoid hyperplasia is characterized by the presence of numerous lymphoid follicles with active germinal centers within thymic medulla [**6**]. This condition is most commonly associated with myasthenia gravis, being seen in up to 65% of cases [**5**].

In contrast, hypertrophic thymus is a CT diagnosis that should be always correlated with patient’s age and clinical context. Hypertrophic thymus can be found in normal patients and does not mean a pathological thymus [**4**].

*Thymomas. *Thymomas are primary epithelial neoplasms that can be completely encapsulated or locally invasive. Encapsulated thymomas are confined within a thick fibrous capsule [**7**].

Thymomas represent 20% of all mediastinal neoplasms in adults; they are the most common anterior mediastinal primary neoplasm in adults but account for less than 5% of mediastinal tumors in children [**8**]. The peak prevalence of thymoma is during the fifth and sixth decades of life; thymomas have no sex predilection [**8**].

Invasive thymomas are histologically identical to encapsulated thymomas but have microscopic evidence of growth outside the tumor capsule [**7**]. Because histologic features of malignancy (i.e., high mitotic activity, prominent nucleoli, and nuclear hyperchromasia) are absent, the term invasive thymoma is preferred over malignant thymoma; invasive thymoma is a separate entity from thymic carcinoma, which is a histologically malignant neoplasm arising from thymic epithelial cells [**7**].

On CT scans, thymomas are generally seen as homogeneous, soft-tissue masses located in the antero-superior mediastinum, closely by the root of the aorta and pulmonary artery, usually projects to one side of the mediastinum. Thymomas vary in size and can have smooth or lobulated borders [**4**]. The mass may be partially or completely outlined by fat or may completely replace the mediastinal fat from the antero-superior compartment. The absence of fatty planes between the thymic mass and mediastinal structures does not necessarily denote the presence of invasion. On contrast enhanced CT scans, the mass enhances homogeneously, unless necrosis and hemorrhage are present [**7**].

The areas of decreased attenuation may correspond to cystic changes. Calcification within a thymoma may be detected on plain radiographs, but are better visualized on CT examination. The pattern of calcification is commonly linear, thin, and peripheral and corresponds to calcium deposition in the tumor capsule. Calcified foci may also be seen scattered throughout the tumor [**7,9**].

With increasing age, the thymus present an progressive process of fatty involution, which makes the detection of thymoma easier in patients over 40 years of age; in contrast, detection of small thymomas in younger patients with residual thymic tissue may be difficult with CT [**7,9**].

Irregular borders between the mass and the adjacent lung suggest the presence of invasion [**7**].

CT has a very high sensitivity for the detection of involvement of the adjacent lung and in the evaluation of pleural and extrapleural seeding by the tumor.

## Conclusions

CT exam is the imaging method of choice following standard chest radiography when thymic pathology is suspected especially in the assessment of patients with myasthenia gravis.

We propose the following CT entities concerning thymic space: normal findings, aspect related to patients’ age (includes remaining thymic mass, remnant of thymic tissue, fatty infiltration of the thymus), diffuse enlargement of the thymus and thymic focal mass.

Although CT is useful in differentiating non-invasive from invasive thymomas, it has a limited value in differentiating thymomas from lymphoid follicular hyperplasia. Sometimes, CT may suggest the histologic nature of a thymic lesion.

## References

[1] Baron RL, Lee JKT, Sagel SS, Peterson RR (1982). Computed Tomography of the Normal Thymus. Radiology.

[2] Fisher ER, Good RA, Gabrielson AE (1964). Pathology of the thymus and its relation to human disease - The thymus in immunology.

[3] Tecce PM, Fishman EK, Kuhlman JE (1994). CT evaluation of the anterior mediastinum: spectrum of disease. RadioGraphics.

[4] Popa GA, Lupescu IG, Georgescu SA The thymus in myasthenic patients: CT- pathologic correlation.

[5] Nishino M, Ashiku SK, Kocher ON, Thurer RL, Boiselle PM, Hatabu H (2006). The Thymus: A Comprehensive Review. RadioGraphics.

[6] Nicolaou S, Muller NL, Li DKB, Oger JJF (1996). Thymus in Myasthenia Gravis: Comparison of CT and Pathologic Findings and Clinical Outcome after Thymectomy. Radiology.

[7] Rosado-de-Christenson ML, Galobardes J, Moran CA (1992). Thymoma: Radiologic-Pathologic Correlation. RadioGraphics.

[8] Nasseri F, Eftekhari F (2010). Clinical and Radiologic Review of the Normal and Abnormal Thymus: Pearls and Pitfalls. RadioGraphics.

[9] Santana L, Givica A, Camacho C (2002). Best Cases from the AFIP: Thymoma. RadioGraphics.

